# Addressing COVID-19 challenges in a randomised controlled trial on exercise interventions in a high-risk population

**DOI:** 10.1186/s12877-021-02232-8

**Published:** 2021-05-01

**Authors:** G. S. Kienle, P. Werthmann, B. Grotejohann, T. Hundhammer, C. Schmoor, Ch Stumpe, S. Voigt-Radloff, R. Huber

**Affiliations:** 1 Centre for Complementary Medicine, Institute for Infection Prevention and Hospital Epidemiology, Medical Centre – University of Freiburg, Faculty of Medicine, University of Freiburg, Freiburg, Germany; 2 IFAEMM at the University of Witten/Herdecke, Freiburg, Germany; 3 Clinical Trials Unit, Medical Centre – University of Freiburg, Faculty of Medicine, University of Freiburg, Freiburg, Germany; 4 Eurythmy4you.com, Nidau, Switzerland; 5 Shen Men Institute, Institute for Qigong, Taiji, Acupressure & Traditional Chinese Medicine (TCM), Düsseldorf, Germany; 6 Centre for Geriatric Medicine and Gerontology Freiburg, Medical Centre – University of Freiburg, Faculty of Medicine, University of Freiburg, Freiburg, Germany; 7 Institute for Evidence in Medicine (for Cochrane Germany Foundation), Medical Centre - University of Freiburg, Faculty of Medicine, University of Freiburg, Freiburg, Germany

**Keywords:** Telemedicine, E-health, Exercise, Trials, Aged, Falls, Tai Chi, Eurythmy therapy, Chronic disease

## Abstract

**Background:**

The coronavirus disease 2019 (COVID-19) pandemic is a threat to ongoing clinical trials necessitating regular face-to-face, in-person meetings, particularly in participants with a high risk of complications. Guidance on how to handle and safely continue such trials is lacking. Chronically ill elderly individuals require—in addition to protection from infection—regular physical exercise and social contact to remain healthy. Solutions on how to handle these conflicting necessities are needed.

The ENTAIER-randomised controlled trial was investigating the influence of mindful movements on fall risk, fear of falling, mobility, balance, life quality, and other outcomes. The study population was planned to comprise of 550 chronically ill elderly individuals with a high risk of falling. The movements were regularly performed in coached groups over 6 months. After the trial began, COVID-19 lockdowns stopped all in-person meetings, and it was expected that the limitations of this pandemic would continue for a long term. Therefore, the exercise programme, which involved complex movements and was typically conducted face-to-face in groups, had to be substituted by a telemedicine programme within a short timeframe. The objectives, therefore, were to identify challenges and tasks that could to be resolved and steps that could to be taken to achieve high-quality, efficacy, safety, and enable human encounter and motivation.

**Methods:**

We proceeded with four steps: 1) A literature review on the quality and feasibility issues of telemedicine in general, and specifically, in exercise training in elderly individuals. 2) Participation in two international telemedicine task forces on integrative medicine, particularly, mind–body medicine. 3) Interviews with study therapists, (for practical purposes, eurythmy therapists and Tai Chi teachers are summarized here as therapists) personnel, and international experts on providing mindful movement exercises and other physiotherapies via live telecommunication technology, and with scientists and patient representatives. 4) Final evaluation by the core trial team and subsequent planning and implementation of changes in the trial organisation.

**Results:**

Various tasks and challenges were identified: for the technical equipment for therapists and patients; for the ability of therapists and trial participants to adequately manage the technology and telemedicine intervention; the reservations and concerns about the technology among therapists and participants; safety and data protection in using the technology; and study design. The two major options found on how to continue the trial in the COVID-19 situation were a complete switch to telemedicine and a partial switch in the form of risk management implemented into the former design.

**Conclusions:**

The management of an ongoing clinical trial in a national or international crisis with a minimum of available time and extra financial resources, alongside with two checklists on steps and procedures for trial continuation and telemedicine implementation, may be informative for other researchers or healthcare providers faced with similar challenges and making similar decisions in the current situation or similar future scenarios.

**Trail registration:**

www.drks.de. DRKS00016609. Registered July 30, 2019.

## Background

*Coronavirus disease 2019* (COVID-19), which is caused by *severe acute respiratory syndrome coronavirus 2* (SARS-CoV-2), was first identified in China at the end of 2019 and then rapidly spread to Europe, the US, and throughout the world. The World Health Organization (WHO) declared the SARS-CoV-2 outbreak a Public Health Emergency of International Concern on 30 January and a pandemic on 11 March. By that time, Europe had become the epicentre of the pandemic [1]. Until an effective vaccination is broadly implemented, precautions should be taken to protect the population, particularly people with a higher risk of infection. Direct person-to-person transmission, mainly via respiratory droplets or aerosols, is the primary route of transmission [2, 3]. Therefore, combining physical distancing, mask use, and hand hygiene measures in the community is the most effective prevention strategy.

SARS-CoV-2 infection can induce several degrees of disease, from asymptomatic to severe, critical, or lethal disease in few patients. Severe illness predominantly occurs in adults with advanced age or underlying medical comorbidities [2]. This patient group is advised to take precautions, practice social distancing, and stay at home [4]. However, limiting physical and social activities and becoming house-ridden impair physical fitness and can accelerate frailty, dependency, and the feeling of isolation, thereby increasing the risk of physical and mental diseases and impairment of chronic diseases. Consistent physical exercise and social activity are essential to maintain physical and emotional health and autonomy and to reduce the risk of falling [5, 6].

Clinical trials, particularly those investigating physical exercise on-site and in-person in elderly individuals, are facing several challenges during the COVID-19 pandemic and the concomitant need for protection of the vulnerable. Answers to these challenges should be found within a short timeframe, as trials and patient care are ongoing and may not be interrupted for a longer period, with unclear prospects on how the pandemic and restrictions will develop and how the compliance of the population with protection measures will be. Furthermore, usually, no or only little additional personal resources are available within the specific trial budget for managing a crisis and the time may not allow for an extra grant application. Guidance on managing clinical trials during this public health crisis has been released by the European Medicines Agency (EMA) [7, 8] and other international bodies [9]. However, many obstacles and questions regarding the application of high-quality treatment and protocol-compliant clinical trials while protecting participants remain unresolved.

A possible solution lies in telemedicine, which started being rapidly and globally implemented. However, the development of telemedicine in exercise interventions, particularly in the elderly, is still in its infancy. We found no guiding principles on how to switch large ongoing clinical trials investigating physical exercise in a high-risk population from in-person to telemedicine interventions and how to adapt these trials to the restrictions introduced by the pandemic.

### The ENTAIER trial

The ENTAIER trial is an ongoing three-armed, multicentre randomised clinical trial [10], which investigates the efficacy and safety of slow, complex, and mindful movement exercises in chronically ill elderly individuals (≥ 65 years) with a high risk of falling. It is registered in the *German Clinical Trials Register* DRKS00016609 (30/07/2019)), funded by the Federal Ministry of Education and Research (BMBF 01GL1805), and has ethics approval in all trial sites (Freiburg: 183/19; 23.07.2019 and 13.07.2020, Tübingen: 561/2019BO1, 21.10.2019 and 12.08.2020, Witten/Herdecke: 150/2019, 31.07.2019 and 30.07.2020, Ulm: 284/19, 01.08.2019 and 22.07.2020, Essen: 19–8908-BO, 18.02.2020 and 23.07.2020; Berlin relies on the vote from Freiburg). At eight trial sites, altogether, 550 outpatients were planned to be randomly assigned to practice either eurythmy therapy (EYT) or Tai Chi for 6 months in small groups (a 4–6 participants, guided by a therapist) and also at home (supported by oral, written, and video instructions) or to receive standard care alone. The primary outcome is fall risk over 6 months. The secondary outcomes were fear of falling, balance, mobility, cognition, mood, life quality, instrumental activities of daily living, use of medical and non-medical services, and adherence, which were assessed at 3, 6, and 12 months.

### The challenges

After initiation of three of the eight study sites from August 2019 on, and after randomisation of 99 of 550 participants into 18 groups, meaning 12 groups regularly practice together, the COVID-19 pandemic interrupted patient recruitment, study site initiations, group sessions, and visits. The intensity and duration of the COVID-19 pandemic were unpredictable but expected to necessitate precautions and restrictions in the following 2 years. These restrictions were expected to vary across the different study sites. They particularly affected the vulnerable population of chronically ill elderly individuals, some of whom were deeply scared by the situation.

### The needs

A solution for this situation had to be found, ensuring the continuity of the trial and its procedures, alongside the care of participants. The solution needed to be adaptable to different pandemic scenarios; applicable at all study sites with different pandemic regulations, authorities, and ethical committees; acceptable for roughly 70 collaborating scientists, clinicians, study personnel, and therapists; and instilling confidence in participants and their relatives so that they could continue to participate despite an unfavourable development regarding the pandemic. Telemedicine (i.e., live, synchronous, two-way video conferencing) was new in mindful and complex exercises, particularly in elderly individuals with a high risk of falling. These exercises were usually taught in-person, and direct face-to-face human interaction was considered essential.

Therefore, our objectives were as follows:

- How to change trial procedures to continue the trial, i.e. the interventions, visits and patient recruitments.
- What protection measures have to be installed for patients, therapists, and study personnel.
- Can a telemedicine-based exercise intervention (via live-streaming internet conference systems) for fall prevention in elderly with a high risk of falling be conducted and be efficacious, safe, and acceptable for the elderly?
- If yes, what challenges and barriers should be addressed, what technological, practical, supportive, communicative and motivational, ethical and scientific measures should be installed to continue with the trial, to preserve the potential efficacy of the intervention, ensure safety, support telemedicine-reluctant and inexperienced elderly participants, and enable human encounters and motivation?

The timeframe for finding answers was very small, and a complete interruption of the trial risked to be the final end due.

Herein, we report on how we proceeded and found guiding principles for substituting the exercise programme with a telemedicine programme during the pandemic and how to adapt the programme to the different abilities, weaknesses, needs, and reservations of the participants. Managing an ongoing clinical trial in such national and international crises and identifying adequate steps and procedures for trial continuation may be informative to other research teams in similar situations.

## Methods

To answer these objectives, the standard methodology would be systematic reviews, consensus-based methods, and piloting if no trials and surveys have yet been conducted. However, due to time pressure and the high complexity of the challenges, time-consuming methods were not possible (even rapid reviews require longer timeframes for each objective [11]). Vast shortcuts were needed, but methodological guidance was unavailable. Therefore, we adapted methodological aspects from the principles of evidence-based medicine, consensus-based guideline development [12], and qualitative research [13, 14].

We conducted the following four steps (April and May 2020):

1. *A literature review on ethical and feasibility issues, and guiding principles for telemedicine in general and specifically, in exercise training in elderly individuals:* [15–40] The literature was searched in PubMed/Medline, Cochrane Database of Systematic Reviews, and Cochrane Central Register of Controlled Trials with the following search terms (as well as hyphenated): “telemedicine”, “telerehabilitation”, “tele-exercise”, “telehealth”, and “eHealth”. The literature was prioritised: consensus- and evidence-based guidelines, other guidelines, guidance, or key discussions from expert groups (e.g. task forces, medical, or physiotherapy societies, ethics or legal experts), systematic reviews, surveys, clinical trials, and trial protocols. Guidelines and guidance were searched for the application of telemedicine in general (not restricted to the elderly), with a stronger focus on exercise. Systematic reviews, surveys, clinical trials, and trial protocols were focused on exercise, telemedicine, and elderly, fall prevention and balance. Language was restricted to English and German. References from key articles were checked. Timeframe was not restricted, but more weight was given to publications from the last five or at most, 10 years. Titles and abstracts were read, and the full article was retrieved if the topic referred directly to our objectives.
2. *Participation in two multidisciplinary international telemedicine task forces on integrative, particularly, mind-body medicine*: One author (GSK) was a member of the task force from the *Society of Integrative Oncology*, with expertise in evidence-based guideline development and collaborating with the *American Society of Clinical Oncology*. It developed guidance for the challenges and barriers of telemedicine care in integrative oncology [41] based on a literature review and a broad survey of approximately 50 integrative oncology experts from 19 countries. Two authors (GSK, PW) were members of the second task force for telemedicine use in Anthroposophic Medicine [42], with a strong focus on EYT, art therapy, and nursing.
3. *In-depth discussions with participating therapists and study personnel of the ENTAIER trial and 11 international experts:* The interviewed experts were purposively sampled [13, 14] to include different perspectives and the following expertise: providing EYT or Tai Chi using telemedicine; providing physiotherapy in high-aged patients with risk of falling; conducting clinical trials in the elderly population and/or on mind–body medicine; applying methods of evidence-based medicine, i.e., systematic reviews and critical assessments of trials, consensus methods, qualitative research; hygiene and patient protection; communication; and patient perspective. Experts were known or recommended to us by experts from the field. The Medical Centre from the University of Freiburg, particularly the Clinical Trial Units in Freiburg, provided additional know-how in the legal requirements, good clinical practice, ethical and data safety issues, data management and statistics. The experts were individually interviewed by one or two trial members (GSK, PW) via phone or video call, and the core trial team met in groups. The procedure was stepwise, meaning that the results and topics from the literature review and from the first interviews were included in subsequent discussions with the experts.

Literature search and expert discussions started with practical objectives: whether a switch to telemedicine is possible and sensible in principle for the trial population; which challenges and barriers should be addressed; which possible solutions and procedures should be taken; and which experiences, evidence, and expert judgements exist. Experts were also asked for comments regarding potential solutions that had already been suggested by other experts and regarding our general proceedings. These were all merged into a table and then discussed with the next experts and checked with results and discussions from literature. Data collection was terminated when no new areas of information or aspects could be captured from the literature or from the experts regarding our objectives [13].

4) *Tabulating and final discussion:* The results were listed in a table, evaluated, and discussed with the core trial team, including patient representatives and a hygiene expert regarding new developments in the COVID-19 pandemic.

## Results

1. The literature on telemedicine issues was vast, and a great variety of different approaches and technologies had been described. Prior studies showed that live, synchronous, online interventions were feasible, effective and safe, and well received by participants and therapists, and enable human encounters. Still, experiences in chronically ill elderly individuals with a high risk of falling were limited. Guidance, recommendations, and discussions referred mostly to more general topics regarding ethical and legal issues, privacy, security, data safety, reimbursement, liability, cultural sensitivity, competences, attitude and skills of providers, communication with and support of the users, confidence, satisfaction and adherence, how to ensure continuity of care, and specific aspects of care and assessments. Some also provide practical recommendations and quick tips for conducting telemedicine services and for developing telemedicine programmes [5, 6, 15–33, 35–40].
2. The integrative medicine online task force dealt very practically with procedural, technical, and ethical issues.
3. The interviews with the experts centred on specific challenges and barriers, alongside potential solutions and steps for the ENTAIER trial. They delivered practical recommendations on all phases of the trial and the interventions and organisation, reflecting the specific expertise of the respective interviewee. The experts also commented on the potential solutions found in the literature, prior interviews, and our whole process of risk adjustment.

The different sources complemented each other, as their details and focus reflected different perspectives and goals. Retrieved solutions and answers did not contradict each other, except two patient representatives who clearly argued against a sole telemedicine solution (see below).

The main challenges and tasks identified from the literature and the interviews were related to issues with: a) technology for therapists and participants; b) the ability of therapists and participants, particularly, if inexperienced, to handle the technique and media-mediated interventions; c) expected reservations and concerns about the technique among some therapists and participants; d) safety and data protection in handling technology.

Altogether, two major solutions were identified and developed: a complete switch to telemedicine elements and risk management implemented in the primary design.

### Complete switch to telemedicine

The advantage of telemedicine is its independence from locally varying developments during the pandemic and its respective varying regulations.

To ensure high quality, safety, feasibility, and adaptation to the abilities of the elderly, several challenges and tasks, alongside possible solutions, were identified and summarised (Table 1). They have implications for study design, study sites, and study organisation in addition to general trial adaptions (e.g. visits), as recommended by EMA [8] (Table 2).

**Table 1:** Technology, Readiness and Safety – Tasks, Challenges, Solutions and Steps for a Complete Switch to Telemedicine in the ENTAIER-Trial

Issue	Task, Challenge	Solution, Steps
Technology for therapists	Older people have limitations in seeing, hearing, and processing information, and special requirements are necessary for the technical equipment used by therapists.	- High resolution streaming camera. - Good illumination in the therapist’s room, with 6–8 light sources (softboxes) and no shadows. - Bluetooth headset. - Sufficient space so the therapist can step back far enough and still be seen from head to toe without distractions in the room; room wide and deep enough so sidewalls are not visible. - Clothing that allows the movements to be seen in high-contrast and does not match the room colour; no distraction due to other items (e.g. plants). - Additional screens that allow the therapist to clearly see the patients. - Support by a second person at least in the first 3–5 h. - Due to investment in equipment and training, possibly only 3–4 therapists per arm, central facilities used by several individuals, or rental of appropriately equipped rooms.
Technology for participants	Older people are only partly equipped with smart electronic devices with video capabilities suitable for video-conferencing (e.g. computer, laptop, or tablet with microphone, speakers, and webcam).	- Recommend support and supply of technical equipment from relatives (e.g. children or grandchildren), neighbours, or friends. - Rental tablets for participants who do not have a smart electronic device. - Pre-setting the tablet.
	Elderly individuals may not have access to reliable internet or Wi-Fi.	- Rental tablet equipped with a SIM card that allows internet access. - Adaption of inclusion criteria.
Handling the technique & method - therapists	Little telemedical experience.	- Training therapists for 1 day with the target population and with the therapist as a test patient to have his or her own experience. - Therapists should know each other and regularly communicate.
	The focus of teaching shifting from primarily visual to primarily verbal instructions.	- Therapists need to be able to teach with a verbal rather than a purely visual teaching style.
	Digital perception of the patient and his or her strengths, limitations, risks, personality is impaired compared to in-person meetings.	- Prior digital consultation of each patient or consultation during the first 1–3 group sessions. - Additional screens (see above).
Handling the technique - participants	Little experience and competence with internet, video-conferences, mobile devices, or desktop computer.	- Train children, grandchildren, or neighbours to assist and support participants with the use of internet, video-conferences, and computers. - Easy-to-use and intuitive online meeting tool. - Good instruction given to participants at the study centre verbally, written, and step-by-step. - Telephone support.
	The interventions are complex and new for participants; the exercises and instructions in the video-conference may not be as easy to understand as in an in-person meeting; individual adaptation may not be as precise; the written manual and audio-visual instructions initially supplied to the participants may be too demanding, especially in the beginning.	- Start slow. Easy instructions. Ask questions for understanding. Record every group session (the therapist), and provide the video for 1 week or until the next lesson (in addition to the written manual and the audio-visual instructions given to the participants).
Reservations about the technique by therapists	Emotional reservations, concerns regarding human encounters, and spiritual aspects.	- Specifically addressing the topic and related sub-aspects, exchanging among therapists and the trial team, lectures by experts trusted as authorities, and training. - Research question for qualitative sub-study and focus group.
	Negative attitude towards the medium may induce nocebo effects.	- Therapists must have a positive attitude towards the medium. - Instructions for positive communication through a communication coach. Put participants at ease when they feel insecure about technology or some technical problems arise. Create a pleasant atmosphere.
	Disappointment of therapists who cannot participate.	- Discussions with therapists who are already included, explaining the current situation of the COVID-19 pandemic.
Reservations about the technique by participants	Emotional reservations and human aspects.	- Assisted test video call with familiar persons (e.g. grandchild or child) during the baseline visit to the study centre. - Explanation of the possibility to stay connected with close ones during lock-down, quarantine, etc. - Communication suggestion for therapists to approach the patient, create a pleasant atmosphere, convey empathy, paraphrase questions, summarise the response, ask about participants‘feelings and experiences with video-conferences, and ask for feedback.
Safety of participants	Participants are at risk of falling and possibly fragile; multitasking is difficult for them.	- Safe space (size, no trip hazards, and stable chair) and checklist for safety aspects. - Instructing children, grandchildren, neighbours, or friends to assist, train, and supervise in the first 3 h. - Clear view of the participants (technical equipment) and clear instructions by therapists (verbal teaching style). - Therapists need to estimate the ability of participants and the group to multitask and, if necessary, rearrange the training so that participants initially only watch the exercise and subsequently perform the exercise without looking at the screen. Train therapists for this situation.
	Sufficient space	- Adapt the inclusion criteria for sufficient space. - Individual adaption of exercises. - Start each class by checking the room for trip hazards.
	Are videos and manuals still suitable (e.g. for security)?	- Check the necessity for additional videos and instructions and of recording therapist instructions.
Data safety	During the video-conferences, other participants and the therapists can see patients and the private room of the patients (and record it if this is intended). Data protection or safety issues of the provider.	- Inform the participants to arrange their clothing and the section in the apartment seen in the conference, accordingly. - Promote privacy and confidentiality. - Investigate the data protection or safety issues of the provider. Consult a data protection officer.

**Table 2:** Implementation of Telemedicine into the ongoing ENTAiER-Trial – Tasks for the Study Team

Issue	Necessity, Challenge	Solution, Task
**Study design**	Additional questions regarding - Feasibility (technology). - Practicability (users). - Satisfaction and acceptance (users). - Barriers (users and technology). - Interaction of patients and therapists. - Suitability of and improvement in the medium for therapists. - Technology and aspects of humanity and confidence. - The degree to which the therapist and participants connect during the exercises or are restrained by the technique.	- Adaptions in trial protocol and patient information. - Additional questions (quantitative analysis). - Possible change in the primary objective. - Qualitative substudy. - Focus group. - Potential filming of a subgroup for analysis. - Informed consent of the subgroup. - Adaptions in the statistical analysis.
**Study centres**	- Increased participant need for information. - Little experience with media.	- Detailed information for participants, necessitating more time; repeat of information when distributing the study book. - Discussion of hurdles, fears, and barriers; if necessary, acquaintance with the media (e.g. test video call with a family member or friend during the baseline visit). - Technical instructions (must be repeated); for some participants, telephone support before the first group session. - Loan tablet, if necessary. - Instruction of assistance at home.
**Study organisation**	- Study organisation, coordination, and management: Planning, organisation or all items, communication with all parties, adaptation of all trial documents, questionnaires, protocols, guidance, ethical approval, discussion with sponsor, adaptation according to EMA recommendations [7].	

#### Estimation and recommendation by public and patient representatives

One of the public and patient representatives supported the necessity of a complete switch to telemedicine. Two others strongly recommended against it, although they saw the necessity of identifying alternatives regarding COVID-19. They reported increasing signs of introversion, concern, and anger among elderly individuals. They saw a dominating telemedicine approach driving passivity, avoidance, and fear, and they stressed the importance of switching to a paradigm based on the elderly’s needs, autonomy, self-responsibility, and joint decision making. Increasing isolation would imply “social death”. The general COVID-19 situation had forced the elderly to become house-ridden and to have television or other digital activities as the main activities, increasing the risk of social isolation and physical and emotional inactivity. This tendency could be enhanced by adding a further telemedicine tool. The patient representatives regarded direct human encounters as essential for the life and internal strength of a person. Therefore, they recommended in-person meetings and information, including hygiene concepts, which should be evidence-based and not fear-driven. This strategy should enable the elderly to participate in society and life again.

### Telemedicine as risk management—the decision for the ENTAIER trial

We discussed the option of a complete switch of the ENTAIER trial to telemedicine within the study organising team and reassessed the situation of COVID-19 in Germany. A total switch to telemedicine would have enabled group exercise, providing motivation for exercise and maintaining human encounters to minimise feelings of isolation without the risk of infection and the necessity to wear masks. However, there was agreement that regarding efficacy, safety, and human encounters, physical meetings for exercise are clearly preferable. Exercises are better to perceive, understand, and follow when there is in-person instruction and interaction compared to instructions seen only on a small digital screen, and exercises can be better adapted to an individual’s strengths and weaknesses. Physical meetings improve human connection and motivation to practise exercise. Furthermore, the effort to attend physical meetings with public or private transport may be an additional activating factor, and absolute infection prevention is only achievable with long-term isolation, which may not be in the patient’s best interest, as specified out by patient representatives. Thus, the goal was to avoid an increase in the normal risk when participating in social activities and following the recommended precautions.

At the time of the evaluation (May 2020), the COVID-19 situation in Germany entered a phase of improvement and stabilisation and seemed to be well handled by the German population and its political and medical authorities. Consequently, the lockdown was reduced, and physical meetings were allowed when hygiene precautions and physical distancing were followed. Thus, physical meetings were less risky, and people increasingly demanded that they return to their normal lives, and elderly individuals wanted to participate in social activities. The acceptance of and expected compliance with mere telemedical intervention were expected to decrease. Therefore, we reconsidered the situation and decided to proceed with the implementation of risk management, i.e. conducting the trial as before but with the implementation of protective measures and with telemedicine options when needed:

Protective measures to reduce the transmission of infection followed the regulations and advice of national and local governments, health authorities, institutions, and relevant guidelines (e.g. social distancing, masks, hygiene, and home isolation after contact with individuals infected with SARS-CoV-2 or when symptoms are present). The risk management strategy evolved from the above listed solutions but was simplified as necessary, considering that the majority of therapy sessions and, particularly, the introductory group sessions, could be held in-person. However, if due to the pandemic, meetings would not be possible, group classes could switch to telemedicine. Participants, particularly, those who are unfamiliar with computers and tablets, or internet-based video conferencing, were encouraged to seek support from family members or friends (e.g. test video calls to friends or family) or will be supported by study personnel in advance to familiarise them with the technology and to reduce stress if and when the use of technology would become necessary. Participants in the Tai Chi or EYT group sessions, who did not have their own computer or tablet, were offered to borrow a tablet from the trial site free of charge.

A user-friendly internet communication system (e.g. whereby.com or Jitsi Meet), which does not require registration or entry of any personal data of the participants, was provided for the groups. Participants were provided with step-by-step written guidance. Organisational precautions were taken so that when social distancing on a national or regional level would become necessary again, group classes could then immediately be switched to telemedicine courses (live, synchronous, internet-based video-conferencing or, if not possible despite all efforts, with instructions via telephone) led by the corresponding Tai Chi teacher or EYT therapist. Participants will then be familiar with the exercises, trainings, and therapists, and hence, can switch to telemedicine classes more easily. In addition, the therapists would have met with the participants, assessed their strengths and weaknesses and could guide and tailor the exercises to them. In case social distancing on an individual level will be necessary (e.g. quarantine or individual concern), patients across the trial sites will be offered participation in group sessions of their assigned intervention using telemedicine. These sessions will be taught by a therapist with additional expertise in telemedicine and the appropriate technical infrastructure. It will be documented whether the session was conducted face-to-face, as a video conference, with verbal telephone instruction only, or not at all. The informed consent form is supplemented with an explanation of the telemedicine risk adjustment. Therapists were instructed on the general and technical details of telemedicine, including positive verbal communication to avoid a nocebo effect; they were offered support with information and training. The study sites were informed about the procedure and how to pass on information and support to the study participants.

When study site visits are not temporarily possible due to COVID-19, the assessments will be performed via telephone and mail. The Berg Balance Scale and the Montreal Cognitive Assessment will then be postponed or performed using telemedicine if the patient is accompanied by a person to assist them and to assure their safety.

The entirety of this risk adjustment was appreciated by patient representatives and was approved by the local ethical committee in Freiburg and at all other study sites. All methods and procedures were performed following relevant guidelines and regulations [10].

### Statistical considerations

As a result of the necessary risk management, it may happen that patients will not attend face-to-face group classes as originally planned but will attend internet-based video-conferences, receive instructions via telephone, or receive no teaching at all. The primary analysis of the trial was planned to be performed in all randomised patients regardless of the intensity or type of Tai Chi and EYT teaching, which addresses the so-called treatment policy estimand according to the recent ICH E9(R1) guideline [43]. Consequently, the effect of the mixture of these interventions will be estimated. The size of the expected effect of this mixture compared to the effect of the originally planned face-to-face group classes can only be speculated, but presumably it will be somewhat smaller. To assess the impact of the restrictions enforced due to the COVID-19 pandemic on the estimated treatment effects, various additional sensitivity analyses were planned. The intensity of the training in each randomised group will be described by the number and type of executed training sessions compared to the originally planned number of training sessions. Sensitivity analyses will be performed, restricting the population to patients who attended at least 80% of the originally planned face-to-face training sessions. Additional analyses will be used to assess how the risk of falls may have been affected by changing the type of teaching. The aim of the ENTAIER trial is to estimate the effects of Tai Chi and EYT training on the risk of falling over 6 months without the influence of the COVID-19 pandemic. Therefore, a treatment effect that is not confounded by pandemic-related disruptions should ideally be addressed. Regarding the ICH E9(R1) guideline [43], the pandemic will be regarded as an intercurrent event, and, in addition to the treatment policy estimand, the hypothetical estimand will be addressed, i.e. a scenario in which the intercurrent event would not occur will be assumed (i.e. addressing the hypothetical estimand). A sensitivity analysis estimating a hypothetical estimand will be performed by disregarding data after face-to-face group classes due to pandemic-related restrictions for an individual patient are stopped. Further sensitivity analyses are conceivable and will be described at a later time in the statistical analysis plan, as the duration of the pandemic and its implications for the course of the study are currently unknown. EMA’s points to consider on the implications of COVID-19 in the methodological aspects of the ongoing clinical trials [8] and the recommendations given by Meyer et al. [44] will be considered.

## Discussion

The COVID-19 pandemic introduced restrictions on medical care and clinical trials. Continuity in care and clinical trials is essential and must be guaranteed to prevent any unnecessary health detriment. Thus, the development of e-health, telemedicine, and tele-rehabilitation has accelerated at an extraordinary speed. Guidance and practical tips have been published as well as technical and feasibility issues, ethical and data safety issues, practical issues, clinical trials, and trial protocols [15–33, 35–40]. Collectively, telemedicine encompasses various conditions, populations, and applications and seems to be similar to in-person care [19, 22–24, 26, 27, 35]. Participants and caregivers feel comfortable and are satisfied in the short term, even if they have no prior experience in virtual participation [27, 39, 40]. However, many questions remain open regarding high-quality, effective, safe, and motivating mindful exercises provided with telemedicine and ensuring human encounters, particularly in chronically ill elderly individuals.

Switching an ongoing trial to telemedicine due to a national or international crisis is particularly challenging, especially in a vulnerable population, chronically ill elderly individuals with a high fall risk, who exercise mindful and complex movements. Such trials need rapid responses, quick decisions, and more specific and tailored adaptions and solutions than are available in published guidance. Literature provides an international framework, covers a wide spectrum and multiple views, evidence, guidance, examples from similar projects and discussions. Assisted by the literature and discussions with international, multidisciplinary experts, we found answers and solutions that enabled us to continue with the ENTAIER-trial, provide transparency, reliability, and an operational framework for the study sites, participants, and their families, study personnel, therapists, and others associated.

Our methodology and our list of challenges, tasks, possible solutions, and steps for the switch to telemedicine (Tables 1 and 2) may support other researchers and practitioners who are in similar situations. The identified issues of technology, readiness to use his technology and safety of using the technology, may apply to many situations of exercise interventions or mind–body medicine that use telemedicine. Therefore, this table could be used as a checklist for other researchers in a similar situation, when introducing new technology in an ongoing trial. Similarly, the tasks for the study team (Table 2) can be used to consider the points to be taken into account for the study management and the organization of the study.

The continuity of physical exercise and social activities during lock-down or quarantine, particularly in elderly individuals, is ultimately critical. A physically inactive lifestyle, social isolation, reduced autonomy, and particularly falls, increase the risk for morbidity and mortality [45–49]. To interrupt this vicious cycle and enable healthy ageing despite chronic disease, it is essential to enhance safe physical, psychological, and cognitive capacities [5, 45, 46, 50]. Many have, therefore, implemented telemedicine exercise or rehabilitation programmes, developed guidance, or are currently testing telemedicine in clinical trials [5, 6, 16, 21, 22, 24, 26, 27, 38–40].

The strengths of our lists (Tables 1 and 2) are the very practical recommendations for handling the specific situations, challenges, and barriers associated with the switch to telemedicine in an ongoing trial in elderly patients. The list focuses on technical issues, emotional reservations, safety, and support of patients and therapists. A strength is also the implementation of several sources from the literature to the perspectives of interprofessional and international experts experienced in clinical research, as well as in the application of different forms of exercise programmes, telemedicine, care of chronically ill elderly individuals with physical and partly mental restrictions, and in mind–body medicine. Another strength has been the cooperation with patient representatives, who are sometimes not heard and often have a different perspective than clinicians and scientists.

There are also limitations to our list. It focused on mindful exercises, chronically ill elderly individuals and the health care situation and population in Germany. Other issues that arise in different types of interventions or other populations or in other countries may not have been covered. In addition, items that had already been included in our clinical trial protocol in detail [10], such as confidentiality, data safety, language barriers, and aspects referring to the details of the intervention, are not listed here. Furthermore, we had limited time for our investigation on how to continue our trial. With more time available, we might have further extended the expert round and included a subsequent Delphi technique. However, experience in telemedicine, even by experts, remains limited and needs to be improved. Therefore, we might not have gained many more aspects with this extended approach, particularly since we had already included a wide range due to the inclusion of aspects from the literature and due to the participation in two telemedicine task forces. Therefore, we presume that we captured most of the important aspects.

## Conclusions

Telemedicine will become increasingly important not only due to the pandemic but also to reaching patients in remote locations or patients who, for other reasons, cannot visit healthcare providers in person. Telemedicine provides several challenges. Therefore, guiding principles, assessments, clinical investigations and key observations must be published. The management of an ongoing clinical trial in a national or international crisis with a minimum of available time and extra financial resources, as well as the identification of adequate steps and procedures for trial continuation, may be informative for other researchers or health care providers faced with similar challenges in the current situation or similar future scenarios. We recommend including public and patient representatives as they have unique and important observations of the group of people who will ultimately benefit from telemedicine. Although this inclusion initially seems time-consuming and costly, it may ultimately accelerate and focus on processes and prevent detours.

## Data Availability

The principle of open data is supported (https://www.bihealth.org/de/quest-center/mission-ansaetze/open-science). Anonymised data may be shared with cooperating scientists or scientists who have other medically or scientifically well-founded reasons (goals referring to transparency, replicability, different analyses, combinations and meta-analyses of data). Their research must aim at improving care of elderly people, and they have to ensure data protection.

## Abbreviations

BMBF: Bundesministerium für Bildung und Forschung (Federal Ministry of Education and Research)
COVID-19: The coronavirus disease 2019
EMA: European Medicines Agency
ENTAIER-trial: Elderly Need Tai Chi and Eurythmy-trial
EYT: Eurythmy therapy
ICH E9(R1). International Council for Harmonisation: Addendum on estimands and sensitivity analysis in clinical trials to the guideline on statistical principles for clinical trials
SARS-CoV-2: Severe acute respiratory syndrome coronavirus 2
